# Polypharmacy and Drug Interaction Risk in Children and Adolescents with Congenital Heart Defects: Insights from a Nationwide Survey

**DOI:** 10.3390/jcm15124802

**Published:** 2026-06-20

**Authors:** Kim Sarah Fritz, Paul C. Helm, Dominik Tobias, Janina Semmler, Jannos Siaplaouras, Christian Apitz, Constanze Pfitzer

**Affiliations:** 1 Deutsches Herzzentrum der Charité, Department of Congenital Heart Disease—Pediatric Cardiology, 13353 Berlin, Germany; 2 Charité—Universitätsmedizin Berlin, Corporate Member of Freie Universität Berlin and Humboldt-Universität zu Berlin, 13353 Berlin, Germany; 3 National Register for Congenital Heart Defects, 13353 Berlin, Germany; 4 Division of Pediatric Cardiology, University Childrens Hospital Ulm, 89075 Ulm, Germany; 5 Department of Obstetrics, Charité—Universitätsmedizin Berlin, Corporate Member of Freie Universität Berlin and Humboldt-Universität zu Berlin, 13353 Berlin, Germany; 6 Pediatrics and Interprofessional Care, University of Applied Sciences Fulda, Health Sciences, 36037 Fulda, Germany; 7 Competence Network for Congenital Heart Defects, 13353 Berlin, Germany

**Keywords:** congenital heart defects (CHD), polypharmacy, drug–drug interaction

## Abstract

**Background**: Congenital heart defects (CHD) are the most common congenital malformations and often require complex, lifelong pharmacotherapy. In pediatric CHD populations, multidrug regimens targeting cardiac function and comorbidities predispose patients to polypharmacy. At the molecular level, concomitant drug use increases the risk of pharmacokinetic and pharmacodynamic interactions. **Methods**: This study aimed to characterize medication patterns and assess polypharmacy and potential drug–drug interactions in patients with CHD. A cross-sectional online survey was conducted in collaboration with the German National Register for Congenital Heart Defects (NRCHD) between November and December 2021. Patients aged 6–17 years with CHD were eligible for inclusion. Participants reported their current medications in open-ended questions. Drugs were categorized into pharmacological classes, and common drug combinations were evaluated for potential interactions. **Results**: Of 894 participants included in the analysis, 372 reported current medication use. Among these, 179 (48.1%) met criteria for polypharmacy (≥2 drugs). Polypharmacy was more frequent in patients with higher disease severity and comorbidity burden. Several drug combinations showed potential for clinically relevant pharmacokinetic and pharmacodynamic interactions, including mechanisms involving renal electrolyte handling, altered protein binding, cytochrome P450-mediated metabolism, and additive pharmacodynamic effects. **Conclusions**: Children with CHD are exposed to complex multidrug regimens with a considerable interaction risk, underscoring the need for systematic medication review and mechanistically informed pharmacological management in pediatric CHD care.

## 1. Introduction

Congenital heart defects (CHD) are the most common type of congenital malformation [1,2]. In Germany, approximately 7000 children are born with CHD each year, and due to major advances in diagnosis and treatment, more than 90% now survive into adulthood [3]. In addition to their underlying cardiac condition, many children and adolescents with CHD are affected by comorbidities and may require repeated medical interventions or hospitalizations [4]. These children are therefore more likely to require ongoing medical care, including interventions and hospitalizations. In addition, physical activity has been shown to be reduced in this population [5]. Taken together, these factors make children with CHD a particularly vulnerable patient group, with an increased likelihood of medication exposure, polypharmacy, and adverse drug reactions [6,7]. This vulnerability is especially relevant in children and adolescents with complex CHD, who often require multiple concurrent therapies, including cardiac medication, treatments for comorbidities, and preventive therapies [6]. However, studies specifically addressing polypharmacy in this population remain limited, although evidence from children with complex chronic conditions and pediatric intensive care settings suggests a substantial medication burden [8]. Furthermore, long-term treatment may lead to persistent polypharmacy beyond childhood and adolescence. Medication management in pediatric patients is additionally complicated by the lack of standardized optimization strategies and by developmental differences in drug absorption, distribution, metabolism, and elimination, which influence both efficacy and side-effect profiles [9]. Together, these aspects highlight an important research gap and underline the need for targeted studies on polypharmacy in children and adolescents with CHD [10].

Beyond the clinical burden of multiple medications, polypharmacy is particularly relevant in children with CHD because concomitant drug use may affect molecular pathways involved in drug absorption, distribution, metabolism, and elimination. Developmental changes in hepatic enzyme activity, renal transporter function, plasma protein binding, and receptor sensitivity can modify both drug exposure and pharmacodynamic response in pediatric patients [10,11]. In children with complex CHD, altered hemodynamics, organ perfusion, cyanosis, and repeated surgical or catheter-based interventions may further influence these mechanisms and increase vulnerability to clinically relevant drug–drug interactions.

A better understanding of medication burden and potential drug–drug interactions in this population is important for optimizing care and improving medication safety. Therefore, this study aimed to provide an overview of self-reported medication use among children and adolescents aged 6–17 years with CHD using data from a nationwide survey conducted in collaboration with the German National Register for Congenital Heart Defects (NRCHD). In addition, selected frequent medication combinations were assessed for potential drug–drug interactions.

## 2. Methods

### 2.1. Study Design

Between November and December 2021, a cross-sectional online survey entitled “E-BAHn” (German acronym for “Ernährung bei Angeborenen Herzfehlern”; English: “Nutrition in CHD patients”) was conducted among patients aged 6–17 years with CHD at the time of survey launch. Participants were recruited via the database of the NRCHD, the largest register for patients with CHD in Europe [12,13]. The survey was conducted between November and December 2021. After data collection, open-ended medication entries required extensive cleaning, manual classification, and plausibility checks before analysis of medication burden and potential drug–drug interactions. The study was conducted in accordance with the Declaration of Helsinki and approved by the institutional ethics committee of Charité—Universitätsmedizin Berlin (protocol code EA2/247/20; approval date: 9 December 2020).

### 2.2. Survey Instruments

As part of the nationwide online survey, participants provided information on their current medication use, including dosing regimens, routes of administration, and therapeutic indications, using open-ended questionnaire items. The questionnaire was completed either by the patient or by a parent/caregiver acting as proxy, depending on the patient’s age and circumstances. In addition to medication-related information, the survey covered respondent type, personal and early-life characteristics, residential and social background, health insurance, physical activity, self-perceived fitness, COVID-19-related changes in activity and nutrition, and previous experiences with nutritional counseling.

### 2.3. Data Based on the National Register for Congenital Heart Defects (NRCHD)

The NRCHD was established in 2003 by German cardiology societies and comprises a nationwide medical database of patients with CHD [13]. More than 60,000 patients and their family members participate in the NRCHD, thereby providing an important basis for clinical and epidemiological research in the field of CHD [3]. Additional clinical information, including primary cardiac diagnosis and history of cardiac surgery or catheter-based intervention, was obtained from the NRCHD. The NRCHD provided registry-based demographic and clinical information, including CHD diagnosis and severity classification. A systematically updated medication list derived from discharge letters, outpatient cardiology visits, general practitioner records, or pharmacy dispensing data was not available for the present survey analysis. Medication use was therefore assessed by self-report.

### 2.4. Data Collection and Classification

**Figure 1:**
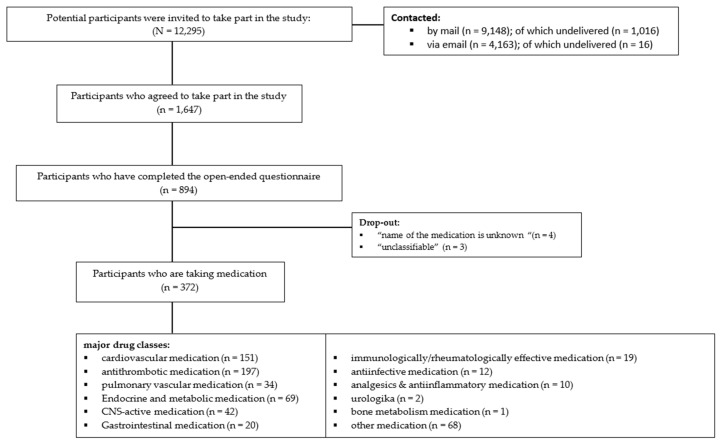
Flowchart of medication data collection and classification.

Self-reported medication entries from open-ended questionnaire fields were manually reviewed and assigned to predefined major drug classes and, where possible, to therapeutic classes shown in Table 1. Ambiguous, incomplete, or unclassifiable entries were handled conservatively and were not used for substance-specific interaction screening unless the active ingredient could be identified with sufficient confidence. Accordingly, if a medication name was missing or unclear or could not be assigned reliably, the entry was categorized as unclassifiable and excluded from class-specific analyses. The overall process of medication data collection and classification is illustrated in Figure 1. For descriptive analyses, the presence of each major drug class was coded as a binary variable (0 = no medication in this class; 1 = medication in this class). Based on the reported medication regimens, the most frequent drug combinations were selected for a structured assessment of potential drug–drug interactions.

### 2.5. Definition of the Term “Polypharmacy”

Polypharmacy was operationally defined as the reported use of two or more medications. This threshold was chosen as a sensitive descriptive indicator of medication burden and potential need for medication review in a pediatric CHD population. It should not be interpreted as evidence of inappropriate prescribing, because multi-drug therapy may be clinically indicated in patients with chronic heart failure, pulmonary hypertension, arrhythmias, complex CHD, or relevant comorbidities.

### 2.6. Statistical Analysis

Owing to the open-ended and descriptive nature of the questionnaire data, analyses were primarily descriptive. Continuous variables are presented as mean ± standard deviation and categorical variables as counts and percentages. Statistical analyses were performed using IBM SPSS Statistics version 28.0 (IBM Corp., Armonk, NY, USA).

### 2.7. AiDKlinik

AiDKlinik (version 4.61.0) is a drug information and prescribing support system developed for use in hospital settings [14]. Selected reported medication combinations were entered into the system to assess potential drug–drug interactions. Interaction severity is displayed within the system using a traffic-light classification.

## 3. Results

### 3.1. Patient Characteristics

Of 1647 patients who consented to participate in the online E-BAHn survey, 894 completed the questionnaire and were included in the analysis (mean age 12.5 ± 3.0 years; 47.2% female). Participants were stratified according to anatomic complexity using the Warnes classification, with 23.8% classified as simple, 37.8% as moderate, and 38.4% as complex CHD [15]. As shown in Table 2, 372 participants reported current medication use, whereas 515 reported no current medication use. A small proportion of responses could not be classified because the medication name was unknown (0.4%) or the entry could not be assigned reliably (0.3%). Among participants receiving medication (n = 372), 179 (48.1%) were exposed to at least two drugs and 40 (10.8%) participants reported taking five or more medications.

### 3.2. Major Drug Group

**Figure 2:**
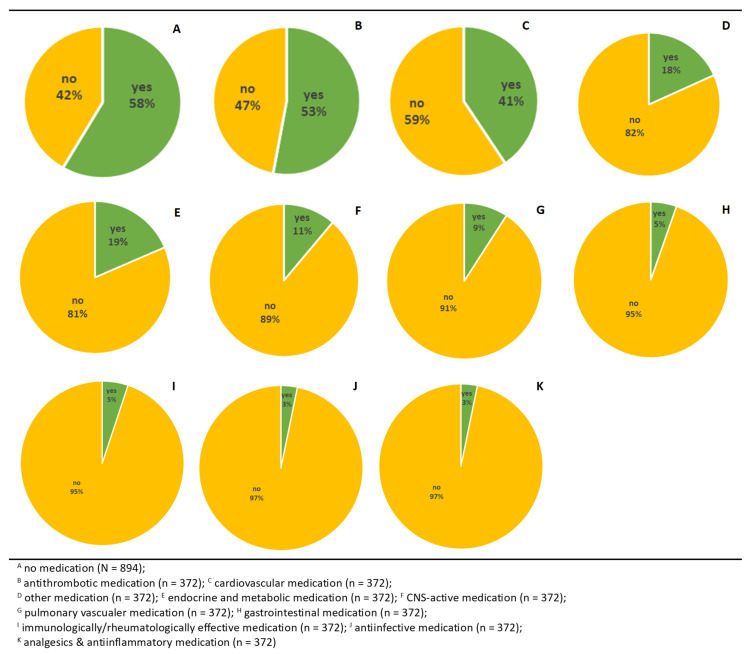
Distribution of major medication classes among participants receiving medication.

**Figure 3:**
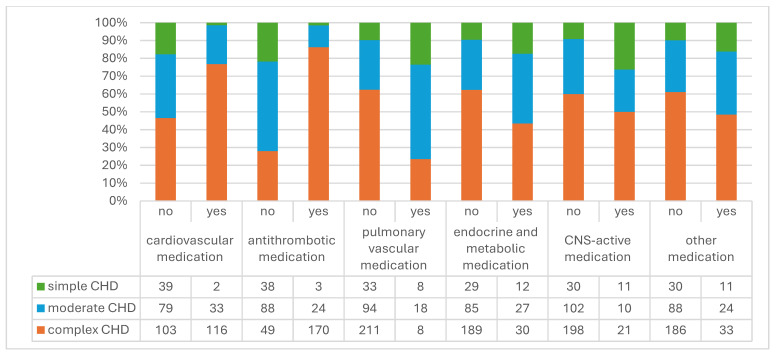
Distribution of the most frequent major medication classes according to CHD severity.

Among participants receiving medication (n = 372), the distribution of major drug classes was examined. The most frequently reported major drug classes were antithrombotic medications (n = 197) and cardiovascular medications (n = 151). Further details are shown in Figure 2. Figure 3 illustrates the distribution of the most frequent major drug classes according to CHD severity.

### 3.3. Cardiovascular Medications

**Figure 4:**
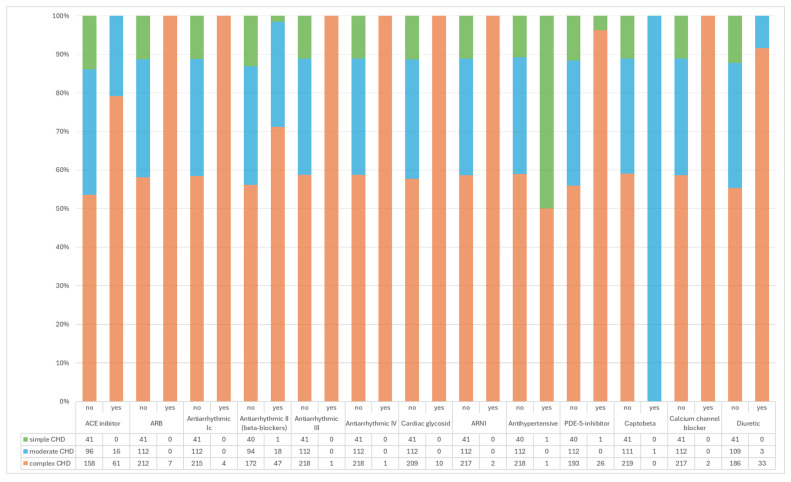
Distribution of cardiovascular therapeutic classes according to CHD severity.

Within the major drug class of cardiovascular medications (n = 151), the most frequently reported therapeutic subgroups were ACE inhibitors (n = 77; 51.0%), beta-blockers (n = 66; 43.7%), and PDE-5 inhibitors (n = 27; 17.9%). The most commonly co-administered major drug classes were antithrombotic medications (n = 94; 25.2%), endocrine and metabolic medications (n = 20; 5.4%), and CNS-active medications (n = 17; 4.6%). As shown in Figure 4, the use of cardiovascular medication appeared to increase with CHD severity.

#### Medication Interaction Check with Cardiovascular Medications

**Figure 5:**
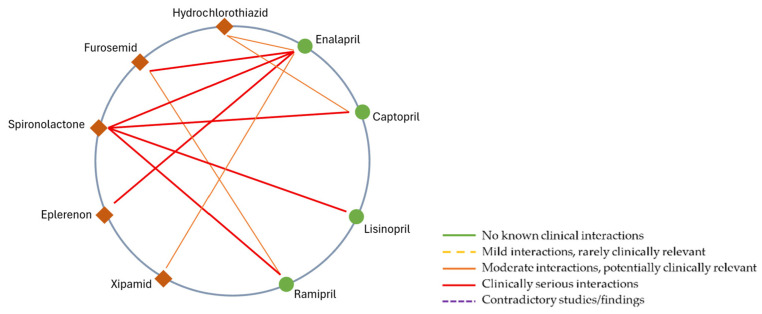
Potential drug–drug interactions within selected cardiovascular medication combinations assessed using AiDKlinik.

Potential drug–drug interactions were identified among selected cardiovascular medication combinations. A total of 18 participants (4.8%) received a combination of ACE inhibitors and diuretics. These combinations were assessed using AiDKlinik. As shown in Figure 5, both moderate and severe potential interactions were identified within this subgroup.

**Figure 6:**
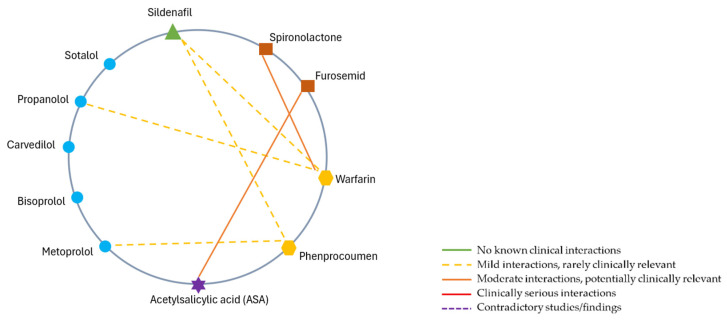
Potential drug–drug interactions between cardiovascular and antithrombotic medications assessed using AiDKlinik.

For further analysis, the three most frequent co-administered major drug classes—antithrombotic medications, endocrine and metabolic medications, and CNS-active medications—were selected. Within these classes, certain therapeutic combinations were particularly common. In the antithrombotic group, cyclooxygenase inhibitors such as acetylsalicylic acid (ASA) were most commonly combined with ACE inhibitors (n = 26; 7.0%), beta-blockers (n = 23; 6.2%), diuretics (n = 13; 3.5%), and PDE-5 inhibitors (n = 7; 1.9%). Among vitamin K antagonists, ACE inhibitors (n = 25; 6.7%), beta-blockers (n = 16; 4.3%), diuretics (n = 17; 4.6%), and PDE-5 inhibitors (n = 14; 3.8%) were the most frequently co-administered cardiovascular therapies. Among these common combinations, only mild and rarely clinically or moderate and potentially clinically relevant interactions were identified (Figure 6).

A total of 17 participants (4.6%) received both cardiovascular and CNS-active medications. Of these, 12 (3.2%) were treated with antiepileptic drugs and 3 (0.8%) with ADHD medication. Among participants receiving cardiovascular and antiepileptic drugs, the therapeutic classes represented included ACE inhibitors (n = 6; 1.6%), diuretics (n = 6; 1.6%) and beta-blockers (n = 3; 0.8%). A severe potential drug–drug interaction involving furosemide and oxcarbazepine was identified among the antiepileptic combinations. Among participants receiving cardiovascular and ADHD medications, the therapeutic classes represented included beta-blockers (n = 2; 0.5%), ACE inhibitors (n = 1; 0.3%), and cardiac glycosides (n = 1; 0.3%). No potential drug–drug interactions were identified within these combinations.

Among participants receiving cardiovascular medications, 11 (3.0%) were also taking thyroid medication. Among those treated with beta-blockers (n = 5; 1.3%), a moderate potential interaction between levothyroxine and metoprolol was identified.

### 3.4. Antithrombotic Medications

**Figure 7:**
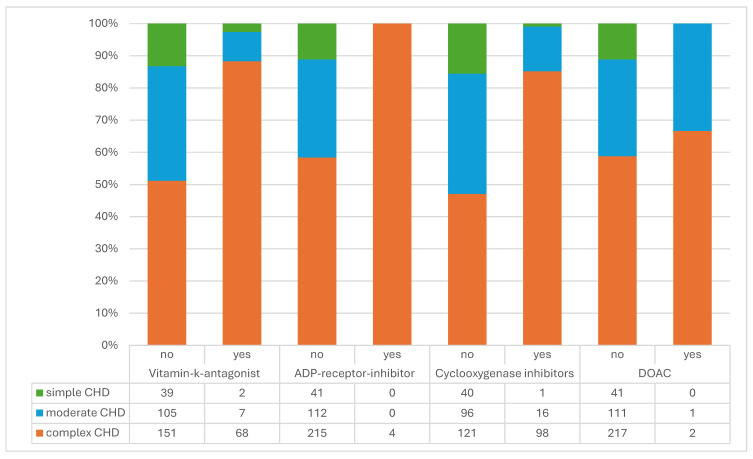
Distribution of antithrombotic therapeutic classes according to CHD severity.

Among the 197 participants receiving antithrombotic medications, the largest subgroup also received cardiovascular medications, as described above. The two other most frequent co-administered major drug classes were CNS-active medications (n = 16; 8.1%) and endocrine and metabolic medications (n = 21; 10.7%). The distribution of antithrombotic medications according to CHD severity is shown in Figure 7 and suggests increasing use with greater CHD complexity.

#### Medication Interaction Check with Antithrombotic Medications

**Figure 8:**
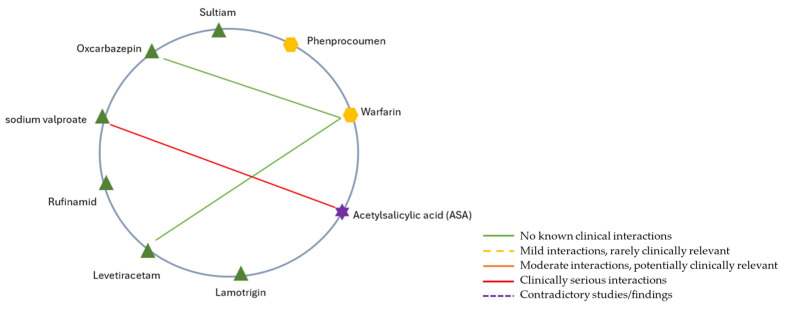
Potential drug–drug interactions between antithrombotic and CNS-active medications assessed using AiDKlinik.

Potential drug–drug interactions were also identified within selected combinations involving antithrombotic medications. In combination with CNS-active medications, a serious potential interaction between antiepileptic drugs and a cyclooxygenase inhibitor was identified (Figure 8). This combination was reported by 7 participants (1.9%).

**Figure 9:**
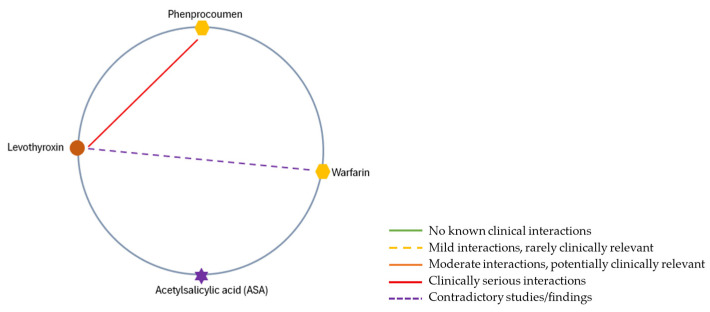
Potential drug–drug interactions between antithrombotic and endocrine/metabolic medications assessed using AiDKlinik.

Potential interactions were also observed in combination with endocrine and metabolic medications. As shown in Figure 9, both serious and contradictory interaction warnings were generated for the combination of vitamin K antagonists and levothyroxine. This combination was reported by six participants (1.6%).

## 4. Discussion

There is currently no uniform definition of polypharmacy in pediatric populations. A scoping review reported that more than 80% of studies defined polypharmacy as the concurrent use of two or more medications [16]. However, in children with chronic conditions, more restrictive definitions, such as the use of five or more medications, are frequently applied to identify patients with a higher medication burden [17,18]. The prevalence of pediatric polypharmacy is highly variable across studies. This variability reflects differences in study populations, outpatient versus inpatient settings, chronic versus short-term medication use, prescription versus self-reported medication sources, observation windows, and thresholds such as two or more versus five or more medications [19]. Consequently, the prevalence estimates reported here should be interpreted in relation to our operational definition and the registry-based survey design. While a threshold of two or more medications is sensitive and may help identify patients who could benefit from medication review, higher thresholds characterize subgroups with greater treatment complexity and medication burden. In pediatric CHD, combination therapy can be guideline- or condition-driven and may reflect appropriate management rather than excessive or inappropriate prescribing. Therefore, medication counts should be interpreted together with diagnosis, indication, cardiac status, and comorbidities.

In this nationwide survey of children and adolescents with CHD, nearly half of the participants receiving medication were exposed to at least two drugs and 40 participants reported taking five or more medications. The results further suggest that medication burden increases with CHD severity and comorbidity burden. Antithrombotic and cardiovascular medications were the most frequently reported major drug classes. Although only selected frequent medication combinations were assessed, potentially clinically relevant drug–drug interactions were identified in a small number of cases.

The most relevant interaction signals in this cohort were observed among cardiovascular medications, particularly combinations involving ACE inhibitors and diuretics. Evidence for this interaction in pediatric populations remains limited. However, data from adult studies suggest that this combination is strongly associated with the occurrence of hyperkalemia [20]. The risk appears to be particularly pronounced in patients with impaired renal function [21]. Therefore, concomitant use should be avoided whenever possible or, if clinically indicated, requires close monitoring and careful benefit–risk assessment [21]. These findings are clinically plausible, as such combinations may increase the risk of electrolyte disturbances, including hyperkalemia, especially in vulnerable patients or in the presence of impaired renal function. Potentially relevant interactions were also identified for combinations involving antithrombotic medication and antiepileptic drugs, as well as vitamin K antagonists and levothyroxine. Although these findings were infrequent, they illustrate that even in a descriptive survey-based cohort, medication regimens with potential clinical relevance can be detected. According to interaction data AidKlinik, ASA—both at anti-inflammatory and low-dose levels—may increase the exposure to free valproic acid by approximately 30–60%, thereby necessitating close clinical monitoring. Although recent randomized studies investigating this interaction are lacking, earlier pharmacokinetic and clinical studies have demonstrated its clinical relevance [22]. More recent literature continues to support the underlying mechanisms, particularly protein-binding displacement, as well as the general susceptibility of valproate to drug–drug interactions [23].

Overall, only a limited number of potentially clinically relevant interactions were identified among the selected combinations assessed in this study. However, this should not be interpreted as evidence of low overall risk. Rather, the present analysis represents an initial overview based on self-reported medication data, and only a subset of medication regimens was systematically screened for interactions. Particularly in participants exposed to multiple medications, more detailed case-by-case evaluation might reveal additional clinically relevant interaction risks. For instance, one patient was prescribed four diuretics concurrently in combination with other medications, and two patients were receiving as many as 12 medications simultaneously, highlighting the complexity of individual treatment regimens and the potential for overlooked interactions. Since the data on medications in this study is based on self-reports, the results should be interpreted as potential indications of interactions derived from these self-reports. Confirmation in clinical practice would require cross-referencing with medical records, pharmacy dispensing data, the prescribed dose and dosing schedule, the duration of treatment, and the clinical context.

In other countries, such as the United Kingdom, clinical pharmacists are integrated into daily ward routines and clinical rounds [24]. Several studies have demonstrated the beneficial impact of clinical pharmacists in pediatric care: A systematic review and meta-analysis reported that pharmacist-led interventions significantly reduce medication error rates in hospitalized children [25]. A randomized controlled trial further suggests improvements in clinical outcomes, including reduced length of hospital stay and enhanced medication management in pediatric inpatients [26]. In addition, an observational study has shown that clinical pharmacists identify a substantial number of drug-related problems, many of which would otherwise remain undetected in routine care [27]. Furthermore, high acceptance rates of pharmacists’ recommendations by physicians underline their clinical relevance and integration into multidisciplinary teams [28]. Recent evidence also highlights their contribution to improving medication safety and interprofessional collaboration in pediatric care settings [29]. For example, a retrospective single-center study from Pakistan examined the role of clinical pharmacists in ensuring safe medication practices in pediatric cardiology in low- and middle-income countries [29]. Dosing errors were identified as the most common problem and were corrected by clinical pharmacists in nearly half of the cases [29]. In addition, the inappropriate use of antibiotics was reduced by approximately 10% [29]. Accordingly, clinical pharmacists can contribute to medication safety in pediatric cardiac care by reviewing medication lists, evaluating indications and dosages, screening for potential drug interactions and supporting medication reconciliation during care transitions and providing medication safety recommendations to families and treating physicians [30]. This role can be particularly important for children and adolescents with congenital heart defects (CHD) who receive combination therapy, have complex comorbidities, or are treated at multiple care facilities.

Germany is among the countries with a high-income level. Therefore, findings from low- or middle-income countries should not be interpreted as directly reflecting the German healthcare context. Nevertheless, such studies illustrate a general principle that is also relevant in resource-rich settings: A structured medication review by clinical pharmacists or clinical pharmacologists can support safe prescribing practices, identify potential drug interactions, and improve medication management in pediatric cardiac patients.

In summary, these findings underscore the importance of structured medication reviews for children and adolescents with CHD. In other healthcare settings, clinical pharmacists are integrated into multidisciplinary care and have been shown to contribute to medication safety, the identification of drug-related problems, and interprofessional collaboration. In pediatric CHD care, such approaches may help to improve medication management, especially in patients with complex disease and high medication burden.

### 4.1. Molecular Mechanisms Underlying Drug–Drug Interactions in Pediatric CHD

From a molecular perspective, the interaction risks observed in this cohort can be grouped into pharmacokinetic and pharmacodynamic mechanisms. Pharmacokinetic interactions alter systemic or tissue exposure by changing absorption, distribution, metabolism, or excretion. In children and adolescents, these processes are not static: maturation of hepatic enzymes, renal filtration and secretion, plasma protein binding, body composition, and transporter expression can modify drug exposure across developmental stages [10,31,32]. Consequently, interaction rules derived from adult populations may not always translate directly to pediatric CHD patients, particularly when cyanosis, heart failure physiology, hepatic congestion, renal impairment, or postoperative states further alter organ perfusion and drug clearance.

Cytochrome P450-mediated metabolism is especially relevant for multidrug regimens. CYP3A4 is involved in the metabolism of a large proportion of clinically used medicines, and recent reviews emphasize that CYP3A activity in children differs from adults because of developmental stage, genetic variability, comedication, diet, and disease state [31]. This is relevant for CHD care because patients may receive cardiovascular drugs together with anti-infective, antiepileptic, endocrine, gastrointestinal, or psychotropic agents. Enzyme induction can reduce exposure and therapeutic efficacy, whereas inhibition can increase exposure and toxicity. In addition, transporter-mediated renal and hepatic clearance may contribute to variability in drug disposition, and pediatric PBPK models increasingly incorporate ontogeny of renal transporters and age-dependent physiology to improve exposure prediction [32,33].

Several interaction signals in the present study are also best explained by pharmacodynamic mechanisms. ACE inhibitor-diuretic combinations may lead to clinically relevant electrolyte and renal effects through converging actions on intravascular volume, renal perfusion, and aldosterone-dependent potassium excretion. Antithrombotic combinations can increase bleeding risk through additive effects on platelet function, coagulation, or anticoagulant exposure. In the case of ASA and valproate, both pharmacokinetic and pharmacodynamic mechanisms may contribute: ASA can displace valproate from plasma protein binding sites, increasing the free pharmacologically active fraction, while concomitant effects on hemostasis may further increase clinical risk [22,23]. Interactions between thyroid hormone replacement and beta-blockers or vitamin K antagonists may reflect changes in adrenergic responsiveness, metabolic rate, and coagulation factor turnover, which are particularly relevant when cardiac reserve is limited.

Mechanistic interpretation is particularly important in pediatric CHD because the same interaction may have different clinical consequences depending on cardiac anatomy, ventricular function, pulmonary vascular disease, renal function, and comorbid neurodevelopmental or endocrine disease. Recent evidence that young patients with CHD exposed to psychotropic medications have increased recurrence of cardiac events further supports the need to consider baseline cardiac vulnerability and pharmacodynamic susceptibility when evaluating comedication [34]. Therefore, medication review in this population should not only identify formal drug–drug interaction warnings but also assess shared molecular pathways, overlapping toxicities, patient-specific organ function, and monitoring parameters such as electrolytes, renal function, INR, heart rate, blood pressure, and electrocardiographic intervals.

### 4.2. Limitations

This study has several limitations. First, because medication data were self-reported, errors in the medication list cannot be excluded. Self-reported medication information may have resulted in incomplete, imprecise, or unclassifiable medication information. In addition, imprecise self-reported information could be the basis of a presumed drug–drug interaction that is not real, for example if an active ingredient was incorrectly reported or inferred. Conversely, omitted medications may have led to underestimation of potential interactions. Incomplete or imprecise information may have led not only to missed potential drug–drug interactions but also to presumed interaction signals that may not reflect actual medication use. Therefore, the interaction analysis should be interpreted as screening for potential drug–drug interaction risk rather than as confirmation of clinically present interactions. Second, the study population was relatively small and may not be fully representative of all children and adolescents with CHD. Third, the analyses were primarily descriptive and only selected frequent medication combinations were assessed for potential drug–drug interactions. Therefore, the reported interaction findings should be interpreted as an initial overview rather than a comprehensive interaction analysis. The drug–drug interaction database used comprises approximately 28,000 documented interactions and is primarily focused on adult populations. Because data were collected in late 2021, the findings should be interpreted as a cross-sectional snapshot of medication use and potential drug–drug interaction risk at that time. Medication patterns, prescribing behavior, clinical recommendations, and the availability or use of digital prescribing-support systems may have changed since data collection. Nevertheless, the present overview highlights the need for further research to identify gaps in care and to explore alternative care structures. Polypharmacy represents a substantial economic burden on healthcare systems, as it is associated with increased healthcare utilization, higher hospitalization rates, and higher direct medical costs [35]. However, structured medication management interventions may partially mitigate these costs [36].

### 4.3. Future Directions

One potential future strategy for medication safety in pediatric CHD could involve structured digital medication lists combined with the continued use of drug information and prescribing support systems such as AiDKlinik. Such tools can facilitate systematic documentation of current medications, automated screening for potential drug interactions, and identification of patients who may require a medication review.

In addition, artificial intelligence and online interaction-checking tools may further improve medication reconciliation and risk stratification in the future. However, these systems should complement, not replace, clinical expertise. The clinical relevance of an interaction warning depends on factors such as dose, treatment duration, indication, comorbidities, renal and hepatic function, monitoring, therapeutic alternatives, and the individual CHD context. Clinical pharmacists and clinical pharmacologists can therefore support the interpretation of digital alerts, reduce alert fatigue, prioritize clinically relevant risks, and translate recommendations into patient-centered care.

Furthermore, future registry-based studies should aim to compare self-reported medication lists with physician-documented medication plans, discharge medications, outpatient records, primary care documentation, and/or pharmacy dispensing data. Such linkage would improve the reliability of medication exposure assessment and drug interaction screening.

## 5. Conclusions

Children and adolescents with CHD represent a vulnerable patient population with a substantial medication burden. The risk of polypharmacy increases with disease severity and comorbidity burden and may be further amplified by hospitalizations and surgical interventions [6,8]. Given the associated risk of clinically relevant drug–drug interactions, structured medication review appears essential to enhance medication safety. Accordingly, digital prescribing support systems and structured medication lists can help identify potential drug interactions more reliably, while the involvement of clinical pharmacists or clinical pharmacologists in the multidisciplinary care of patients with CHD can support the patient-specific interpretation and implementation of medication safety recommendations. In high-risk pediatric CHD patients, particularly those with higher medication burden or complex comorbidities, the routine involvement of clinical pharmacists or clinical pharmacologists in multidisciplinary congenital cardiac care may support medication reconciliation, identification of potential drug–drug interactions, and optimization of pharmacological management. Integrating clinical pharmacists into multidisciplinary care teams may represent an effective strategy to identify potential interactions and optimize pharmacological management in this high-risk population [37].

## Figures and Tables

**Table 1:** Classification of medications into major drug groups and therapeutic classes.

Major Drug Class	Therapeutic Class	
**cardiovascular medication**	ACE inhibitors Angiotensin II receptor blockers (ARB) Antiarrhythmics I–IV Cardiac glycosides Angiotensin receptor—neprilysin inhibitors (ARNI)	Antihypertensives PDE-5-inhibitors Captobeta Calcium channel blocker Diuretics
**antithrombotic medication**	Vitamin K antagonist ADP receptor inhibitor	Cyclooxygenase inhibitor Direct Oral Anticoagulants (DOAC)
**analgesics and anti-inflammatory medication**	Analgesics Aminosalicylates	
**pulmonary vascular medication**	Leukotrien receptor antagonist Endothelin receptor antagonist beta-2 adrenergic agonists	inhaled corticosteroids (ICS) Inhalation solution
**endocrine and metabolic medications**	Antidiabetic drugs Contraceptives Antidiuretics	Thyroid medications Growth hormones Sex hormones Melatonin
**CNS-active medications**	Antiepileptic drugs ADHD medication Antipsychotics	Muscle relaxants Benzodiazepines
**gastrointestinal medication**	Proton pump inhibitors (PPI) Laxatives	Digestive aids Bile and liver remedies
**anti-infective medication**	Antibiotics	
**immunological/rheumatological effective medications**	Antiallergic drugs Antihistamines	Immunosuppressants Immunoglobulins
**bone metabolism**	Bisphosphonates	
**urological medications**	Anticholinergics Antispasmodics	
**other medications**	Supplements Phytopharmaceuticals	Homeopathy Other

**Table 2:** Overview of patient characteristics in the overall study population and in the subgroup receiving medication.

	Full Study Cohort		Study Cohort Receiving Medication	
	(N = 894)		(n = 372)	
**Gender**				
male	472 (52.8%)		224 (60.2%)	
female	422 (47.2%)		148 (39.8%)	
**Age in years**	12.5 ± 3		12.5 ± 3	
**Age group**				
6–11 years	286 (32.0%)		115 (58.6%)	
12–17 years	608 (68.0%)		257 (69.1%)	
**Premature Birth**				
yes	152 (17.0%)		64 (17.2%)	
no	728 (81.4%)		304 (81.7%)	
unknown	14 (1.6%)		4 (1.1%)	
**Primary diagnosis (top 3)**	VSD ^1^	170 (19.0%)	UVH ^2^	124 (33.3%)
	UVH ^2^	134 (15.0%)	VSD ^1^	43 (11.6%)
	TOF ^3^	124 (13.9%)	TOF ^3^	42 (11.3%)
**Secondary diagnosis**	Hypothyroidism	25 (2.8%)	Hypothyroidism	21 (5.6%)
	Epilepsy	11 (1.2%)	Epilepsy	9 (2.4%)
	ADHD ^4^	5 (0.6%)	ADHD ^4^	3 (0.8%)
**Syndromic diseases**				
yes	106 (11.9%)		65 (17.5%)	
no	788 (88.1%)		307 (82.5%)	
**CHD classification**				
simple	213 (23.8%)		41 (11.0%)	
moderate	338 (37.8%)		112 (30.1%)	
complex	343 (38.4%)		219 (58.9%)	
**Surgery for a CHD**				
yes	356 (39.8%)		278 (74.7%)	
no	538 (60.2%)		94 (25.3%)	
**Intervention (IV) for a CHD**				
yes	544 (60.9%)		186 (50.0%)	
no	350 (39.1%)		186 (50.0%)	
**Surgery and IV**				
yes	270 (30.2%)		165 (44.4%)	
no	276 (30.9%)		73 (19.6%)	

^1^ VSD = ventricular septal defect; ^2^ UVH = univentricular heart defect; ^3^ TOF = Tetralogy of Fallot; ^4^ ADHD = attention deficit hyperactivity disorder.

## Data Availability

The original contributions presented in the study are included in the article, further inquiries can be directed to the corresponding author.

## Abbreviations

The following abbreviations are used in this manuscript:

CHD	Congenital heart defects
NRCHD	National Register for Congenital Heart Defects
E-BAHn	German acronym for “Ernährung bei Angeborenen Herzfehlern”, English translation: “Nutrition in CHD patients”
VSD	ventricular septal defect
UVH	univentricular heart defect
TOF	Tetralogy of Fallot
ADHD	attention deficit hyperactivity disorder
ASA	acetylsalicylic acid
